# A Compact Two-Dimensional Varifocal Scanning Imaging Device Actuated by Artificial Muscle Material

**DOI:** 10.3390/biomimetics8010120

**Published:** 2023-03-13

**Authors:** Yang Cheng, Chuanxun Chen, Lin Liu, Jie Cao, Yingying Xu, Qun Hao

**Affiliations:** 1 Key Laboratory of Biomimetic Robots and Systems, Ministry of Education, Beijing Institute of Technology, Beijing 100081, China; ycheng@bit.edu.cn (Y.C.); 3220205080@bit.edu.cn (C.C.); 3220225097@bit.edu.cn (L.L.); caojie@bit.edu.cn (J.C.); 3420205014@bit.edu.cn (Y.X.); 2 Yangtze Delta Region Academy of Beijing Institute of Technology, Jiaxing 314003, China; 3 National Institute of Metrology, Beijing 100029, China

**Keywords:** two-dimensional varifocal scanning, Alvarez lens, decentered lens, artificial muscle material, dielectric elastomer

## Abstract

This paper presents a compact two-dimensional varifocal-scanning imaging device, with the capability of continuously variable focal length and a large scanning range, actuated by artificial muscle material. The varifocal function is realized by the principle of laterally shifting cubic phase masks and the scanning function is achieved by the principle of the decentered lens. One remarkable feature of these two principles is that both are based on the lateral displacements perpendicular to the optical axis. Artificial muscle material is emerging as a good choice of soft actuators capable of high strain, high efficiency, fast response speed, and light weight. Inspired by the artificial muscle, the dielectric elastomer is used as an actuator and produces the lateral displacements of the Alvarez lenses and the decentered lenses. A two-dimensional varifocal scanning imaging device prototype was established and validated through experiments to verify the feasibility of the proposed varifocal-scanning device. The results showed that the focal length variation of the proposed varifocal scanning device is up to 4.65 times higher (31.6 mm/6.8 mm), and the maximum scanning angle was 26.4°. The rise and fall times were 110 ms and 185 ms, respectively. Such a varifocal scanning device studied here has the potential to be used in consumer electronics, endoscopy, and microscopy in the future.

## 1. Introduction

A compact two-dimensional varifocal scanning imaging system, which exhibits the ability to provide detailed object information and adjust the interesting area to make the object centered in the field of view, plays a crucial role in the fields of robots, aerospace, and biomedicine, etc. [1,2,3,4].

Up to now, various methods have been proposed to achieve two-dimensional varifocal scanning imaging. Based on the differences in their operating mechanisms, these methods can be broadly divided into two categories: mechanical and non-mechanical. Mechanical varifocal scanning methods include the microelectromechanical [5,6,7,8], servo motor [9,10,11,12], piezoelectric elements [13,14], manual movement [15], and external force [16]. However, for the mechanical varifocal scanning methods, the focal length variation is small, and the scanning range is difficult to expand [17]. The use of multiple optical components and the requirement of longitudinal movement over them often leads to large system sizes, difficulty to achieve high accuracy, and rapid varifocal scanning [17,18,19]. In addition, it is difficult to insert external optical components into the varifocal scanning systems with limited working distance. A method of manual movement actuation of the varifocal scanning device was proposed [20], but the tuning speed and precision could not be scaled to modern image applications [21,22]. However, the focusing range and speed of varifocal scanning are constrained by the size of the microlenses and the mechanical characteristics of the substrate. Optical-phased array technology is a typical non-mechanical varifocal scanning method [23,24]. Optical-phased array technology has the potential to address some of the issues posed by traditional variable scanning methods. However, the limited processing technology restricts the scanning angle of the optical-phased array technology, resulting in relatively low scanning efficiency [25,26,27]. A confocal scanning device using the Alvarez–Lohmann lens was proposed. This device axially scans volumetric samples, while preserving the locations of the initial point source, as well as that of the detector plane [28]. Therefore, a two-dimensional varifocal scanning element with a compact structure, fast response speed, and large varifocal and scanning angles is highly desirable.

In this paper, we propose a compact two-dimensional varifocal scanning device. The varifocal and scanning functions of the proposed device are realized by the varifocal principle of the Alvarez lenses and the scanning principle of the decentered lens, respectively.

This varifocal concept was rediscovered independently and simultaneously by Alvarez and Lohmann [29,30,31]. Different from the traditional varifocal lens that changes the focal length through the axial shifting of solid lenses, the Alvarez lenses can provide precise and rapid dynamic adjustment of optical power through the lateral displacement of two cubic phase masks [32,33,34]. The Alvarez lenses have recently been regarded as an attractive method to achieve varifocal function rapidly while still maintaining a compact structure [35]. The decentered lens method is a promising option to achieve the scanning function because of its simplicity, which is only composed of two lenses. One remarkable advantage of both the Alvarez lenses and the decentered lenses is that a small displacement perpendicular to the optical axis can realize a large varifocal range and large scanning angle, respectively [36,37]. The traditional methods to actuate the Alvarez lens and the decentered lenses include MEMS-driven units, motor, and manual movement [15]. However, these actuators have some drawbacks, such as small displacement, slow speed, and complex structure, which result in small varifocal and scanning ranges, slow response speed, and bulkiness in size.

Electroactive polymers are a class of materials that exhibit deformation on a large scale under an electric field [38,39]. Within the family of electroactive polymers, dielectric elastomer (DE) is rapidly becoming a preferred choice of soft actuators due to its high strain, energy density, efficiency, response speed, noise-free operation, resilience, and lightweight properties [40]. DE is well known as an ‘artificial muscle’, and it is suitable as an actuator for the application fields of bio-inspired robots, adaptive optics, energy harvesters, etc. Biomimetics is the process of deriving good design from nature. Benefiting from these distinct advantages of the DE, the Alvarez lenses for varifocal function, and decentered lenses for scanning function are both actuated by artificial muscle in this paper.

The rest of the paper is organized as follows: Section 2 describes the principle of the two-dimensional varifocal scanning imaging device actuated by artificial muscle material. Section 3 presents the design and fabrication process of the varifocal scanning element. The experimental results are presented in Section 4, and Section 5 is the conclusions.

## 2. Principle of the Proposed Compact Two-Dimensional Varifocal Scanning Device

**Figure 1:**
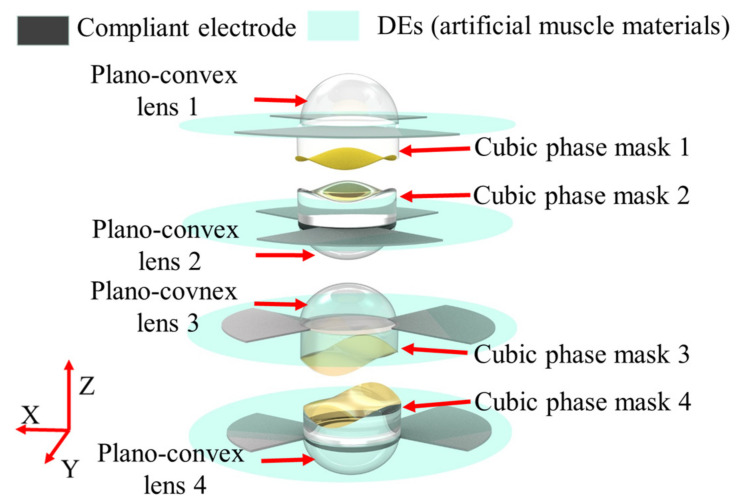
The architecture of the proposed two-dimensional varifocal scanning element.

As shown in Figure 1, the proposed compact two-dimensional varifocal scanning device comprises four identical decentered lenses, two identical Alvarez lenses, four Des (artificial muscle materials), and compliant electrodes. The four decentered lenses have a plano-convex shape, i.e., plano-convex lens 1, plano-convex lens 2, plano-convex lens 3, and plano-convex lens 4. Each of the two Alvarez lenses, i.e., Alvarez lens 1 and Alvarez lens 2, has two cubic phase masks. Alvarez lens 1 is composed of cubic phase mask 1 and cubic phase mask 2. Alvarez lens 2 is composed of cubic phase mask 3, and cubic phase mask 4. Cubic phase masks are arranged in tandem, with free-form surfaces facing each other, which realizes the optical power tuning by slightly shifting relative to each other in a transverse direction relative to the optical axis. The flat surfaces of the cubic phase masks are opposite to the plane surfaces of the corresponding plano-convex lenses and are mounted in the middle area of the four DEs. The four DEs are divided into two quadrants and both sides of the two quadrants are coated with compliant electrodes. The DEs of the cubic phase mask 1, cubic phase mask 2, plano-convex lens 1, and plano-convex lens 2 are coated with compliant electrodes along the *y* directions, and that of the cubic phase mask 3, cubic phase mask 4, plano-convex lens 3, and plano-convex lens 4 are coated with compliant electrodes along the *x* directions.

When applying an actuation voltage across one quadrant of the dielectric elastomer through the compliant electrodes, the Coulomb force between free charges on the electrodes generates Maxwell’s stress induced by the applied electrical potential in the thickness direction. Maxwell’s stress reduces the distance between the compliant electrodes; thus, the dielectric elastomer expands in the lateral directions, because it is an incompressible material. The relationship between the applied voltage (*V*) and the Maxwell pressure (*p*) can be expressed as:

(1) $$p=\varepsilon \varepsilon_{0}{\left(V/d\right)}^{2},$$

where *ε*_0_ and *ε* are the vacuum permittivity and the relative permittivity of the DE, respectively, and d is the thickness of the DE. Since the DE is an incompressible material, Maxwell’s stress makes the DE expands in the lateral directions. The expansion in the lateral direction enables lens elements to undergo a radial uniform squeezing. Therefore, the decentered lenses and the cubic phase masks can be moved in the lateral directions by applying actuation voltage on the compliant electrodes of one quadrant of the DEs. By applying different voltages on the different quadrants, the Alvarez lenses can realize the varifocal function and the decentered lens can realize the scanning function by being moved in the lateral direction.

The varifocal principle based on Alvarez lenses is easily understood. Each cubic phase mask of the Alvarez lens has a plane surface and a free-form surface. The free-form surface is described by a cubic polynomial equation, which can be given by [32,33,34]:

(2) $$t=A(xy^{2}+x^{3}/3)+Dx+E,$$

where *A*, *D*, and *E* are constants to be determined as well as *x*, and *y* is transverse coordinate normal to the *z*-direction, and *t* is the phase profile of the Alvarez lens. Different from the traditional varifocal method that is based on mechanical movement along the optic axis, the Alvarez lenses provide an optical power-tuning range through small lateral displacements, perpendicular to the optical axis. Assuming the lateral displacement is *δ*, the focal length of the Alvarez lens (*f*) can be expressed as:

(3) $$f=\frac{1}{4\delta A(n-1)},$$

where *n* is the refractive index of the Alvarez lens material. The varifocal function can be achieved by applying actuation on the compliant electrodes of the DEs adhered to the four Alvarez lenses, the two-dimensional.

The scanning principle based on decentered lenses is also easily understood. The incoming collimated wavefront is focused to a point in the back focal plane of the first lens, while the second lens is situated so that its front focal plane coincides with the back focal plane of the first lens. The decentered second lens then re-collimates the exiting light, but the beam is directed to a non-zero steering angle. Based on this principle, the decentered lenses provide a view transformation through small lateral displacements perpendicular to the optical axis.

Because the cubic phase masks and the decentered lens can be moved by actuating the DEs, the two-dimensional varifocal scanning function can be achieved. The principle is described as follows: at the initial state, the four cubic phase masks are precisely aligned along the optic axis and the four plano-convex lens centers are aligned with the four cubic phase masks, respectively. The flat surfaces of the cubic phase masks are opposite to the plane surfaces of the corresponding plano-convex lenses. At the actuated state, the compliant electrodes of quadrants of the DEs are subjected to a controllable actuation voltage, for example, when the plano-convex lens 1 and cubic phase mask 1 are moved in opposite directions with plano-convex lens 2 and cubic phase mask 2 in the *x* direction. Therefore, a translated displacement between the plano-convex lens 1 and plano-convex lens 2 is generated, and a translated displacement between the cubic phase mask 1 and cubic phase mask 2 is produced. According to the geometrical optics, when plano-convex lens 1 and plano-convex lens 2 are decentered from the principal optical axis with a translated displacement, objects will be scanned along the *x* direction. When the cubic phase mask 1 and the cubic phase mask 2 are decentered from the principal optical axis with a translated displacement, objects will be magnified or demagnified along the displacement direction. Similarly, varifocal scanning in the *y* direction can be achieved by applying voltages to compliant electrodes of the DEs of the plano-convex lens 3, cubic phase mask 3 and plano-convex lens 4, and cubic phase mask 4. Thus, the two-dimensional varifocal scanning function can be achieved.

**Figure 2:**
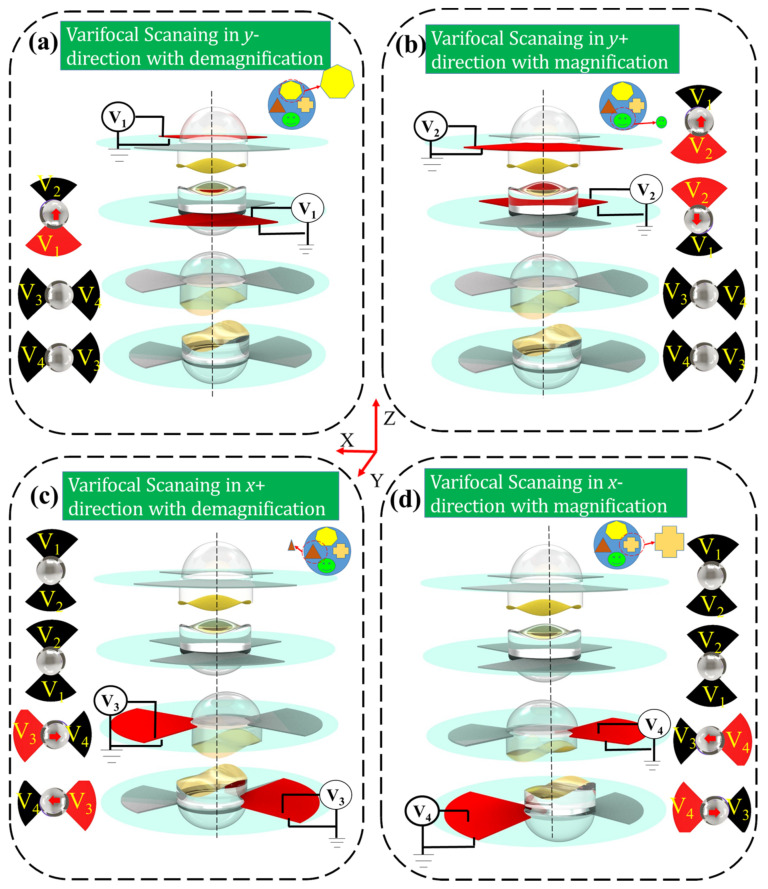
The schematic diagram of the varifocal scanning element, actuated at four varifocal scanning states by applying different voltages on the four DEs. The red areas represent that the compliant electrodes are active. The proposed varifocal scanning device scans the object along the *y*+ direction with magnification capacity (**a**); along the *y*− direction with demagnification capacity (**b**); along the *x*+ direction with demagnification capacity (**c**); and along the *x*− direction with magnification capacity (**d**).

In order to clearly describe the principle of the two-dimensional varifocal scanning device, four varifocal scanning states are shown in Figure 2. The compliant electrodes of four quadrants of the DEs are subjected to four actuation voltages (*V*_1_, *V*_2_, *V*_3_, *V*_4_) to make the four plano-convex lenses and four cubic phase masks move in the two-dimensional direction, to realize varifocal scanning. The red areas represent that the compliant electrodes are active, i.e., the actuation voltage is not zero. As shown in Figure 2a, when the actuation voltage *V*_1_ is active, the plano-convex lens 1 and cubic phase mask 1 move along the *y*+ direction, meanwhile the plano-convex lens 2 and cubic phase mask 2 move along *y*− direction. The active actuation voltage V_1_ makes the proposed varifocal scanning element scan the object in the *y*- direction with demagnification capacity. Similarly, as shown in Figure 2b, when the actuation voltage V_2_ is active, the plano-convex lens 1 and the cubic phase mask 1 move along *y*- direction, meanwhile the plano-convex lens 2 and the cubic phase mask 2 move along *y*+ direction. The actuation voltage *V*_2_ makes the proposed varifocal scanning element scan the object along the *y*+ direction, with magnification capacity. As shown in Figure 2c, the varifocal scanning in the *x*− direction with demagnification capacity can be realized through active actuation voltages of *V*_3_. The varifocal scanning along the *y*+ direction with magnification capacity can be realized through active actuation voltages of *V*_4_, as shown in Figure 2d. Hence, the proposed element has the ability of two-dimensional varifocal scanning by actuating the four DEs.

## 3. Fabrication of the Proposed Two-Dimensional Varifocal Scanning Device

**Figure 3:**
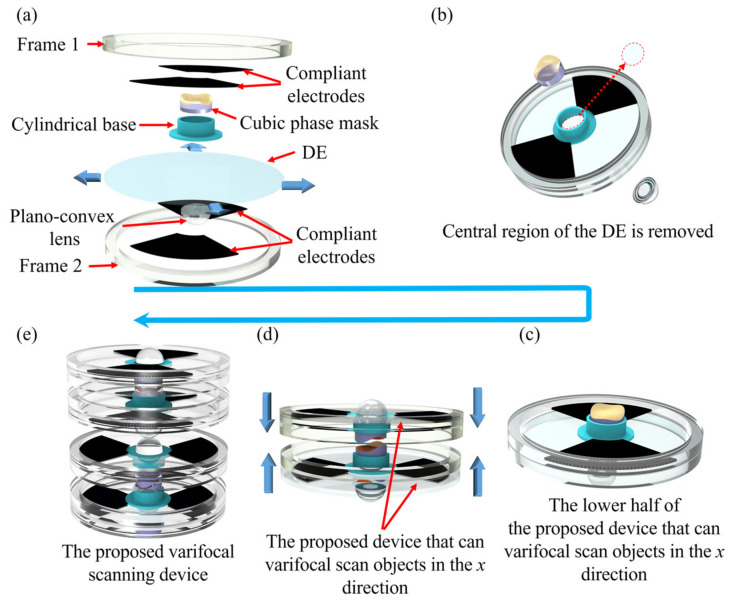
Schematic illustration of the fabrication procedure of the proposed varifocal elements. (**a**) The main components of the varifocal scanning device. The DE (VHB4905, 3M company) is pre-stretched with a ratio of 200%. The top and bottom sides of two local areas of the DE along the *x*-axis are coated with carbon powder (BP2000, Carbot) as compliant electrodes. (**b**) The central region of the DE is removed and replaced by the cylindrical base. (**c**) The lower half of the proposed device can varifocal-scan objects in the *x* direction. (**d**) The proposed device can varifocal-scan objects in the *x* direction. The upper part is the same as the lower part and it is rotated 180°. (**e**) Assembled varifocal scanning device.

The fabrication processes of the proposed varifocal scanning device are described as shown in Figure 3. The structure of the varifocal scanning device includes four plano-convex lenses and four cubic phase masks, eight polymethyl methacrylate (PMMA) frames (inner diameter of 38 mm and outer diameter of 42 mm), four DEs (VHB 4905, 3M Company, Saint Paul, MN, USA), copper foils, and compliant electrodes, as shown in Figure 3a.

From the architecture of the proposed two-dimensional varifocal scanning device, we can find that the eight lenses (four plano-convex lenses and two Alvarez lenses) are the important optical elements. Four commercial 6 mm diameter lenses with a 6 mm focal length (GCL-010130A, Daheng Optics, Beijing, China) were employed as the plano-convex lenses. Concerning the four cubic phase masks, we fabricated them through the diamond-turning and replication molding process [41]. The Alvarez lens material was the UV-curable optical adhesive, NOA83H (Norland, New York, NY, USA), with a refractive index of 1.56. The parameters of the four cubic phase masks were the same, which were A = 0.075 mm^−2^, D = −0.175, and E = 1 mm in Equation (2), respectively.

**Figure 4:**
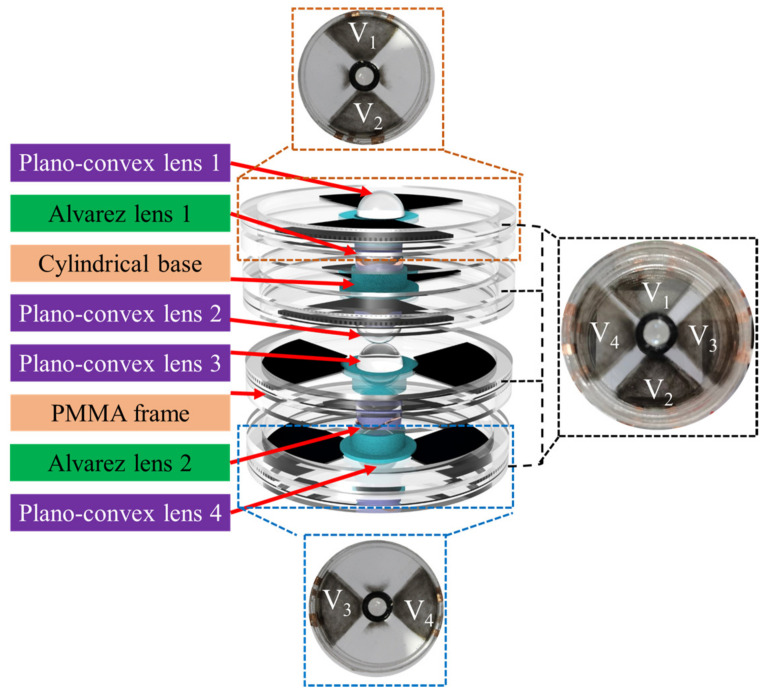
The structure and fabrication of the varifocal scanning device.

Firstly, the eight PMMA frames were fabricated by a laser-engraving machine (4060, Ketailaser Company, Liaocheng, China). As shown in Figure 3a, the DE (VHB4905, 3M Company) was sandwiched by the two PMMA frames, and the top and bottom sides of local areas of the DE along the *x*-axis of the cubic phase masks were coated with carbon powder (BP2000, Carbot, Boston, MA, USA) as compliant electrodes. With the help of self-designed fan-shaped masks, the carbon powder was printed on the two quadrants of the DE by using a brush. The DE was biaxially stretched by a factor of 200% to achieve a large strain performance. Secondly, to eliminate the effect of DEs in the optical path on imaging, a cylindrical base was used to hold the cubic phase mask and the de-centered lens in the center, and the DE under the cylindrical base was removed, as shown in Figure 3b. Because DE (VHB4905) is a kind of strong adhesion tape, the cylindrical base, and the acrylic frames could directly adhere to the VHB4905. The cylindrical base was fabricated using a 3D printer (Raise3D) with a printing accuracy of 0.01 mm. Thirdly, the cubic phase mask was precisely placed into the cylindrical base under a microscope camera (GP-530H, Gaopin Precision Instrument Company, Kunshan, China). The plane of the Alvarez lens faced outside the cylindrical base, and the cubic phase mask faced inside the cylindrical base. The plano-convex lens was also mounted into the cylindrical base and the plane of the plano-convex lens was aligned to the plane of the cubic phase mask. These components were precisely assembled, as shown in Figure 3c. Fourthly, the same components were fabricated and rotated 180° as well as, then combined with the components in Figure 3c, to form the unit of the proposed varifocal scanning device that can varifocal-scan objects in the *x* direction, as shown in Figure 3d. Lastly, the fabrication process of the unit of the varifocal scanning device that can varifocal-scan objects in the *y* direction was the same as that in the *x* direction. The top and bottom sides of the local areas of the DE under the cubic phase mask were coated with carbon powder along the *y*-axis. The cubic phase masks of the unit of the varifocal scanning device that move in the *y* direction could varifocal-scan objects in the *y* direction. The fabricated structure of the proposed two-dimensional varifocal scanning device is shown in Figure 4.

## 4. Experiments and Discussion

**Figure 5:**
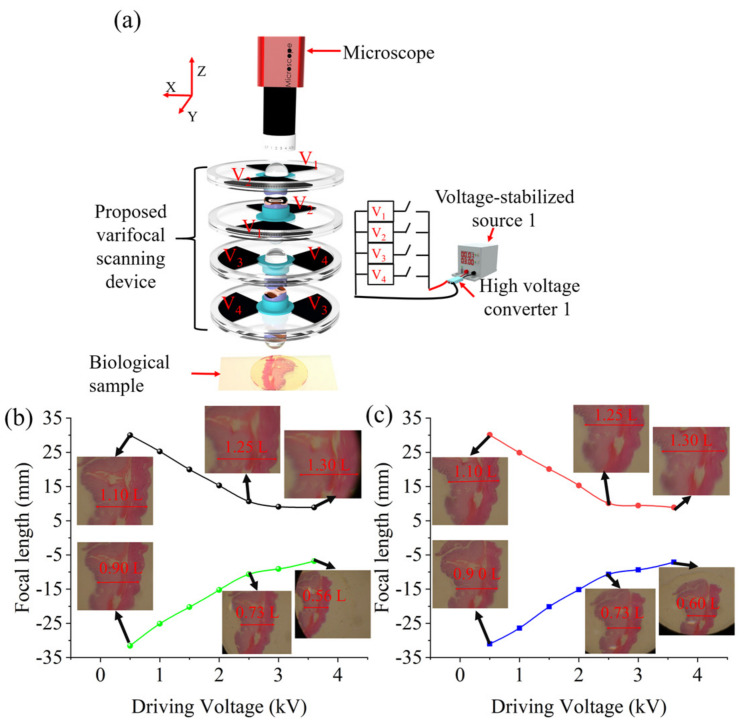
The experimental focal lengths of the varifocal scanning device. The experimental focal length was measured by the magnification method. (**a**) Experimental schematic for evaluating the focal length of the varifocal scanning device. (**b**) The black line indicates that the proposed varifocal scanning device scans the object along *x*+ direction with magnification capacity; the blue line indicates that the proposed varifocal scanning device scans the object along *x*− direction with demagnification capacity; (**c**) the red line indicates that the proposed varifocal scanning device scans the object along *y*+ direction with magnification capacity, and the blue line indicates that the proposed varifocal scanning device scans the object in *y*− direction with demagnification capacity.

The varifocal range is an important parameter to evaluate the varifocal scanning device. To qualitatively assess the varifocal performance, the r focal length of the varifocal scanning device at the four states, shown in Figure 2, was measured by using the magnification method. The experimental schematic is shown in Figure 5a. A biological stomach tissue section was located at a fixed distance of 3.0 mm (*D*) from the proposed varifocal scanning device as the imaging object, and was imaged by the microscope with the proposed varifocal scanning device. By applying an actuation voltage on the compliant electrodes, using a voltage-stabilized source (UTP3315TFL-II, UNI-T Company, Dongguan, China), the Alvarez lenses could magnify the object and the decentered lens allowed for the scanning of the object in different directions, which endowed the proposed device with the capacity of varifocal scanning. By measuring the size of the object in the captured image under different driving voltages, the focal length of the varifocal scanning device was obtained. The driving voltage, generated from the voltage-stabilized source and amplified 1200 times by the high-voltage converter, was applied to the compliant electrodes through copper foils. The focal length (*f*) was calculated by the following equation: *f* = *DM*/(*M* − 1), where *M* is the optical magnification of the object [42]. Focal lengths at the four states shown in Figure 2 were shown in Figure 5b,c (see Video S1 in Supplementary Materials). From Figure 5b, we can find that the focal length decreased from 30.1 mm to 8.9 mm (the black line), with an increase in the driving voltage from 0 kV to 3.6 kV when the proposed varifocal scanning device scans the object along *x*+ direction with magnification capacity. On the other hand, the focal length decreased from −31.6 mm to −6.8 mm (the green line), with an increase in the driving voltage from 0 kV to 3.6 kV when the proposed varifocal scanning element scans the object along the *x*− direction with demagnification capacity. From Figure 5c, we can also find that the focal length decreased from 30.1 mm to 8.9 mm (the red line), with an increase in the driving voltage from 0 kV to 4 kV when the proposed varifocal scanning device scans the object along *y*+ direction with magnification capacity. The focal length decreased from −31.0 mm to −7.1 mm (the blue line), with an increase in the driving voltage from 0 kV to 3.6 kV when the proposed varifocal scanning device scans the object along the *y*− direction with demagnification capacity. Therefore, the focal length variation of the proposed varifocal scanning device was up to 4.65 times (31.6 mm/6.8 mm).

**Figure 6:**
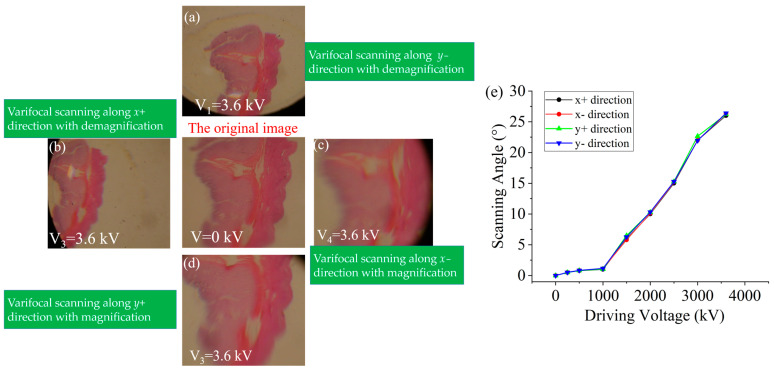
(**a**–**d**) The imaging results of the proposed varifocal scanning device under four varifocal scanning states through applying actuation. (**e**) The relationship between the scanning angle and the driving voltage under four varifocal scanning states.

The range of scanning is also an important parameter to evaluate the varifocal scanning device. We experimentally measured the varifocal scanning range of the proposed device. A biological stomach section was selected as the imaging object. The distance between the object and the varifocal scanning device was 3 mm (*D*). Under different actuation voltages on the different quadrants, the varifocal scanning images of the object are shown in Figure 6. From Figure 6, we can find that the object was scanned in two-dimensional directions, including the *x*-axis (left and right), and the *y*-axis (top and bottom) directions. The scanning angle was calculated from the displacement distance (*l*) of the object over the center of the field of view by a simple triangle function, i.e., arctan (*l*/*D*). The scanning angle of the device under different applied voltages is shown in Figure 6e. The scanning angle increased with an increase in the amplitude of the actuation voltages. The displacement of the image was up to 1.5 mm. Therefore, the maximum scanning angle was calculated to be approximately 26.4° under the actuation voltage of 3.6 kV. The results showed that the scanning angle of the device was slightly different when the same voltages were applied to different quadrants of the DEs. This maybe can be dedicated to the measuring error, the misalignments of the Alvarez lenses and the decentered lenses, and the uniform pre-stretch of the DEs.

**Figure 7:**
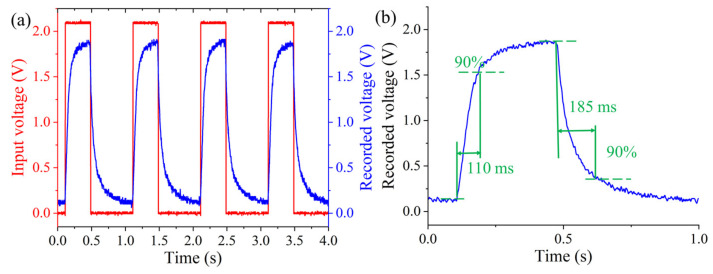
Dynamic response of the proposed varifocal scanning device. (**a**) The recorded voltage of the photodetector when the input voltage was a square signal, with a frequency of 1 Hz and an amplitude of 2.0 V. (**b**) The measured rise time and fall time of the proposed varifocal scanning device. The rise and fall times were 110 ms and 185 ms, respectively.

The response speed for the varifocal scanning device is an important parameter to evaluate the dynamic performance. The response speed was also tested. A green laser beam (λ = 532 nm) was generated by a laser (MGL-III-532, New Industries Optoelectronics Technology Company, Changchun, China) and focused on the photodetector (PDA36A-EC, Thorlabs, Newton, NJ, USA) through the proposed varifocal scanning device. The beam was collimated by a beam expander (GCO02501, Daheng Optics, Beijing, China) and passed through a diaphragm with a 5 μm pinhole (GCT-060201, Daheng Optics, Beijing, China) to eliminate stray light. The square wave signal, with a period of 1 s, a peak-to-peak amplitude of 2.0 V, and a duty cycle of 30%, was amplified by a power amplifier (PA1011, RIGOL Technologies, Suzhou, China) and then is applied to the compliant electrode (*V*_1_) of the DEs to change the focal length. The variable focal length made the recorded light intensity change and then the recorded voltage different. The experimental result is shown in Figure 7. The time of rise and fall was regarded as the time consumption from the initially recorded voltage to 90% of the maximum recorded voltage and from the maximum recorded voltage to 90% of the initially recorded voltage [43]. The response time of the proposed varifocal scanning device was obtained from the local magnified area in Figure 7a. From Figure 7b, it can be observed that the rise and fall times of such a device were 110 ms and 185 ms, respectively. The response time can be further decreased using the lens material with low density, and DEs with a high Young’s modulus.

## 5. Conclusions

In summary, we present here a novel two-dimensional varifocal scanning device. The proposed varifocal scanning device can both change the focal length continuously and scan the object in a two-dimension direction. The varifocal function of this proposed device is realized by the principle of laterally shifting cubic phase masks, and the scanning function is realized by the principle of decentered lenses. The varifocal function and the scanning function were actuated by artificial muscle material (DEs). The focal length variation of the proposed varifocal scanning device was up to 4.65 times higher, where the maximum focal length was 31.6 mm and the minimum focal length was 6.8 mm. The two-dimensional scanning angle of the proposed varifocal scanning device was up to 26.4°. The response time was tested and the results showed that the rise and fall times were 110 ms and 185 ms, respectively.

## Data Availability

Data are available upon request, due to privacy and ethical restrictions.

## Supplementary Materials

The following supporting information can be downloaded at: https://www.mdpi.com/article/10.3390/biomimetics8010120/s1, Video S1: A compact two-dimensional varifocal scanning imaging device, actuated by artificial muscle material.
